# Supplementary material for: A guanosine metabolism-bioenergetics intersection drives Parkinson’s disease
Source: bioRxiv. 2026 Aug 10:2026.07.29.741503. Preprint. [Version 2] doi: 10.64898/2026.07.29.741503 (PMC13640495; doi:10.64898/2026.07.29.741503)
Supplement: Supplementary file 1 [file NIHPP2026.07.29.741503v2-supplement-4.pdf]

Figure S1. Dynole compounds validation

(A) Average vGlutI-pHI traces in 0.1 mM glucose (blue) and 25 µM Dynole 34-2 (pink) incubated acutely, normalized to peak height, where neurons were challenged with 600

bioRxiv preprint doi: https://doi.org/10.64898/2026.07.29.741503; this version posted August 10, 2026. The copyright holder for this preprint (which was not certified by peer review) is the author/funder, who has granted bioRxiv a license to display the preprint in perpetuity. It is made available under a CC-BY 4.0 International license.

APs at 10 Hz (black bar). (B) The remaining vGlutI-pHI fluorescence values were quantified 60 s post stimulation, mean ± SEM, N=9 and 8 respectively, unpaired t-test. Inhibition of the GTPase activity of Dynamin blocks SV endocytosis.

Figure S2. NDK1/2 KD can be suppressed by boosting ATP production

(A) Representative images of neurons expressing mTagBFP2 (yellow) along with NDK1/2 shRNA (cyan arrow) next to non-transfected (grey arrow) and immunostained against NDK1 (cyan) and NDK2 (magenta), scale bar 10 µm. Quantification of the KD efficiency of NDK1 (B) and NDK2 (C), mean ± SEM, paired t-test, N=15 for both (B) and (C). (D) Exocytic extend of the traces from Fig. 1G, ordinary 1-way ANOVA. (E) Average vGlutI-pHI traces in 5 mM glucose (teal) and NDK1/2 KD (grey) normalized to peak height, where neurons were challenged with 600 APs at 10 Hz (black bar). (F) The remaining vGlutI-pHI fluorescence values were quantified 60 s post stimulation, mean ± SEM, N=10 and 7 respectively, unpaired t-test. (G) Average vGlutI-pHI traces in 0.1 mM glucose (blue) and 100 µM Ellagic Acid (grey) added acutely normalized to peak height, where neurons were challenged with 600 APs at 10 Hz (black bar). (H) The remaining vGlutI-pHI fluorescence values were quantified 60 s post stimulation, mean ± SEM, N=9 and 12 respectively, unpaired t-test. Pharmacological inhibition of NDKs, mimics silencing of the enzymes. (I) Average vGlutI-pHI traces in 0.1 mM glucose (blue), NDK1/2 KD (black), NDK1/2 KD plus PGK1-HALO (orange) and NDK1/2 KD plus 10 µM Terazosin (yellow) normalized to peak height, where neurons were challenged with 600 APs at 10 Hz (black bar). (J) The remaining vGlutI-pHI fluorescence values were quantified 60 s post stimulation, mean ± SEM, N=9, 12, 12 and 8 respectively, ordinary 1-way ANOVA. Upregulation of ATP kinetics under hypometabolic conditions suppresses the NDK silencing.

Figure S3. Guanosine can suppresses synaptic dysfunction caused by PARK mutations

(A) Survival analysis of the mRuby-NDK1 (red) and mRuby (blue) animals in Fig. 2A, following the 6-OHDA unilateral injection. NDK1 expression promoted animal survival. (B) Quantification of mRuby and TH double positive neurons from mRuby-NDK1 (red) and mRuby (blue) animals, normalized to the non-lesioned side TH+, mean ± SEM, N=8 and 8 respectively, unpaired t-test. (C) Average vGlutI-pHI traces in 5 mM glucose of wt (light blue), wt + HALO-NDK1 (light red) and wt + 100 µM Guanosine for 2 h (light green) normalized to peak height, where neurons were challenged with 600 APs at 10 Hz (black bar). (D) The remaining vGlutI-pHI fluorescence values were quantified 60 s post stimulation, mean ± SEM, N=8, 11 and 8 respectively, 2-way ANOVA. NDK1 and Guanosine had no effect in wt neurons.

Figure S4. IMPDH1 and 2 KD validates the pharmacological inhibition

bioRxiv preprint doi: https://doi.org/10.64898/2026.07.29.741503; this version posted August 10, 2026. The copyright holder for this preprint (which was not certified by peer review) is the author/funder, who has granted bioRxiv a license to display the preprint in perpetuity. It is made available under a CC-BY 4.0 International license.

(A) Representative images of neurons expressing mTagBFP2 (cyan) along with IMPDH1 shRNA (cyan arrow) next to non-transfected (grey arrow) and immunostained against IMPDH1 (magenta), scale bar 10 µm. (B) Quantification of the KD efficiency of IMPDH1, mean ± SEM, N=7, paired t-test. (C) Representative images of neurons expressing mTagBFP2 (cyan) along with IMPDH2 shRNA (cyan arrow) next to non-transfected (grey arrow) and immunostained against IMPDH2 (magenta), scale bar 10 µm. (D) Quantification of the KD efficiency of IMPDH2, mean ± SEM, N=14, paired t-test. (E) Average vGlutI-pHI traces in 0.1 mM glucose (blue), IMPDH1 KD (black) and IMPDH2 (gray) normalized to peak height, where neurons were challenged with 600 APs at 10 Hz (black bar). (F) The remaining vGlutI-pHI fluorescence values were quantified 60 s post stimulation, mean ± SEM, N=8, 9 and 17 respectively, 2-way ANOVA. Blocking the rate limiting enzymes in guanosine nucleotide de-novo synthesis impacts synaptic transmission. (G) Average vGlutI-pHI traces in 0.1 mM glucose (blue), 2 µM Ribavirin (yellow) and 2 µM Ribavirin + 100 µM Guanosine incubated for 2 h (green) normalized to peak height, where neurons were challenged with 600 APs at 10 Hz (black bar). (H) The remaining vGlutI-pHI fluorescence values were quantified 60 s post stimulation, mean ± SEM, N=9, 15 and 5 respectively, 2-way ANOVA.

Figure S5. Mitochondria are necessary for the BH_4 mediated upregulation of ATP production

(A) Representative images of neurons expressing mTagBFP2 (cyan) along with GCH1 shRNA (cyan arrow) next to non-transfected (grey arrow) and immunostained against GCH1 (magenta), scale bar 10 µm. (B) Quantification of the KD efficiency of GCH1, mean ± SEM, N=11, paired t-test. (C) Average vGlutI-pHI traces in 0.1 mM glucose (blue) and 1 µM SPRi3 incubated ON (orange) normalized to peak height, where neurons were challenged with 600 APs at 10 Hz (black bar). (D) The remaining vGlutI-pHI fluorescence values were quantified 60 s post stimulation, mean ± SEM, N=10 and 16 respectively, unpaired t-test. Blocking SPR, the BH_4 synthetic enzyme, mimics the GCH1 inhibition. (E) Average syn-ATPSnFR2.0-miRFP670nano3 traces in 0.1 mM glucose + 2 µM Oligomycin (blue), GCH1-HALO (red) and 50 µM BH_4 incubated ON (green) normalized to baseline, where neurons were challenged with 600 APs at 10 Hz (black bar). (F) The remaining ATP values were quantified at the end of the stimulation, mean ± SEM, N=9, 9 and 11 respectively, ordinary 1-way ANOVA. The BH_4 mediated upregulation of ATP production required functional mitochondria. (G) Hierarchical sunburst visualization of significant single-gene associations derived from GP2 NBA and WGS datasets across 11 ancestry groups. Analyses were performed under unadjusted and covariate-adjusted models (age, sex, PC1–PC5). FDR–Benjamini–Hochberg correction was used. Intronic and intergenic variants were excluded to focus on coding and regulatory exonic signals. Segment size represents the number of significant associations per category. (H) Graphical summary and proposed mechanism. We had previously identified that PGK1 is the rate-limiting step

bioRxiv preprint doi: https://doi.org/10.64898/2026.07.29.741503; this version posted August 10, 2026. The copyright holder for this preprint (which was not certified by peer review) is the author/funder, who has granted bioRxiv a license to display the preprint in perpetuity. It is made available under a CC-BY 4.0 International license.

in synaptic glycolysis, producing sufficient ATP to power synaptic transmission. In this study, we identify that guanosine nucleotide metabolic pathways have an outsized effect on synaptic transmission. In addition, we unravel NDKs as the enzymes connecting all these pathways and establish their implication in PD, using a pallet of cellular, in-vivo and human genetic approaches.

Table S1. Significant single-variant association results from logistic regression analyses adjusted for sex, age and the first five principal components (PCs). Abbreviations: A1, effect allele; CI, confidence interval; FDR-BH, Benjamini–Hochberg false discovery rate; Freq, frequency; HGVS, Human Genome Variation Society; NBA, NeuroBooster Array; OR, odds ratio; PCs, principal components; WGS, whole-genome

Table S2. Significant gene-based rare variant association results from Sequence Kernel Association Test (SKAT) analyses across ancestry groups and variant classes. Variant classes: Rare variants (MAF < 1%); Potentially Functional, non-intronic variants; Coding, protein-altering coding variants; LoF, predicted gene-disrupting variants; Damaging, missense variants with CADD PHRED ≥ 20; MAF < 0.01% + LoF, loss-of-function variants with MAF < 0.01%; MAF < 0.01% + LoF + Damaging, loss-of-function and damaging missense variants with MAF < 0.01%. Abbreviations: LoF, loss-of-function; MAF, minor allele frequency; N_INFORMATIVE, number of individuals with informative genotype data for the tested region; NumVar, number of variants included in the gene-based test; NumPolyVar, number of polymorphic variants among those included; Q, SKAT test statistic. Abbreviations: AAC, African Admixed; AFR, African; AJ, Ashkenazi Jewish; AMR, American Admixed; CAS, Central Asian; EAS, East Asian; EUR, European; FIN, Finnish; MDE, Middle Eastern; SAS, South Asian; CAH; Complex Admixture.

Table S3. Significant gene-based rare variant association results from Sequence Kernel Association Test–Optimal (SKAT-O) analyses across ancestry groups and variant classes. Variant classes: Rare variants (MAF < 1%); Potentially Functional, non-intronic variants; Coding, protein-altering coding variants; LoF, predicted gene-disrupting variants; Damaging, missense variants with CADD PHRED ≥ 20; MAF < 0.01% + LoF, loss-of-function variants with MAF < 0.01%; MAF < 0.01% + LoF + Damaging, loss-of-function and damaging missense variants with MAF < 0.01%. Abbreviations: LoF, loss-of-function; MAF, minor allele frequency; N_INFORMATIVE, number of individuals with informative genotype data for the tested region; NumVar, number of variants included in the gene-based test; NumPolyVar, number of polymorphic variants among those included; Q, SKAT-O test statistic; rho, SKAT-O mixing parameter. Abbreviations: AAC, African Admixed; AFR, African; AJ, Ashkenazi Jewish; AMR, American Admixed; CAS, Central Asian; EAS, East Asian; EUR, European; FIN, Finnish; MDE, Middle Eastern; SAS, South Asian; CAH; Complex Admixture.

bioRxiv preprint doi: https://doi.org/10.64898/2026.07.29.741503; this version posted August 10, 2026. The copyright holder for this
(which was not certified by peer review) is the author/funder, who has granted bioRxiv a license to display the preprint in perpetuity. I
available under a CC-BY 4.0 International license.

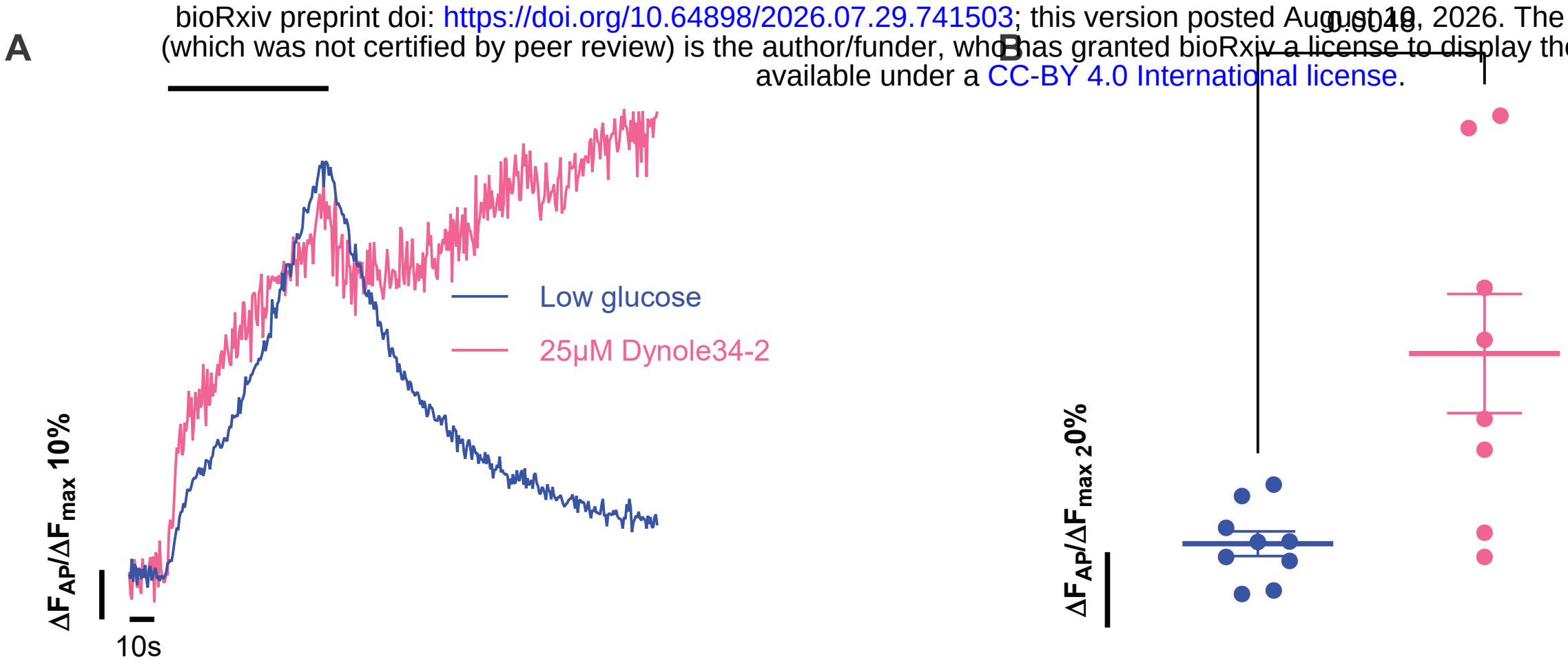

Figure S1

bioRxiv preprint doi: https://doi.org/10.64898/2026.07.29.741503; this version posted August 10, 2026. The copyright holder for this preprint (which was not certified by peer review) is the author/funder, who has granted bioRxiv a license to display the preprint in perpetuity. It is made available under a CC-BY 4.0 International license.

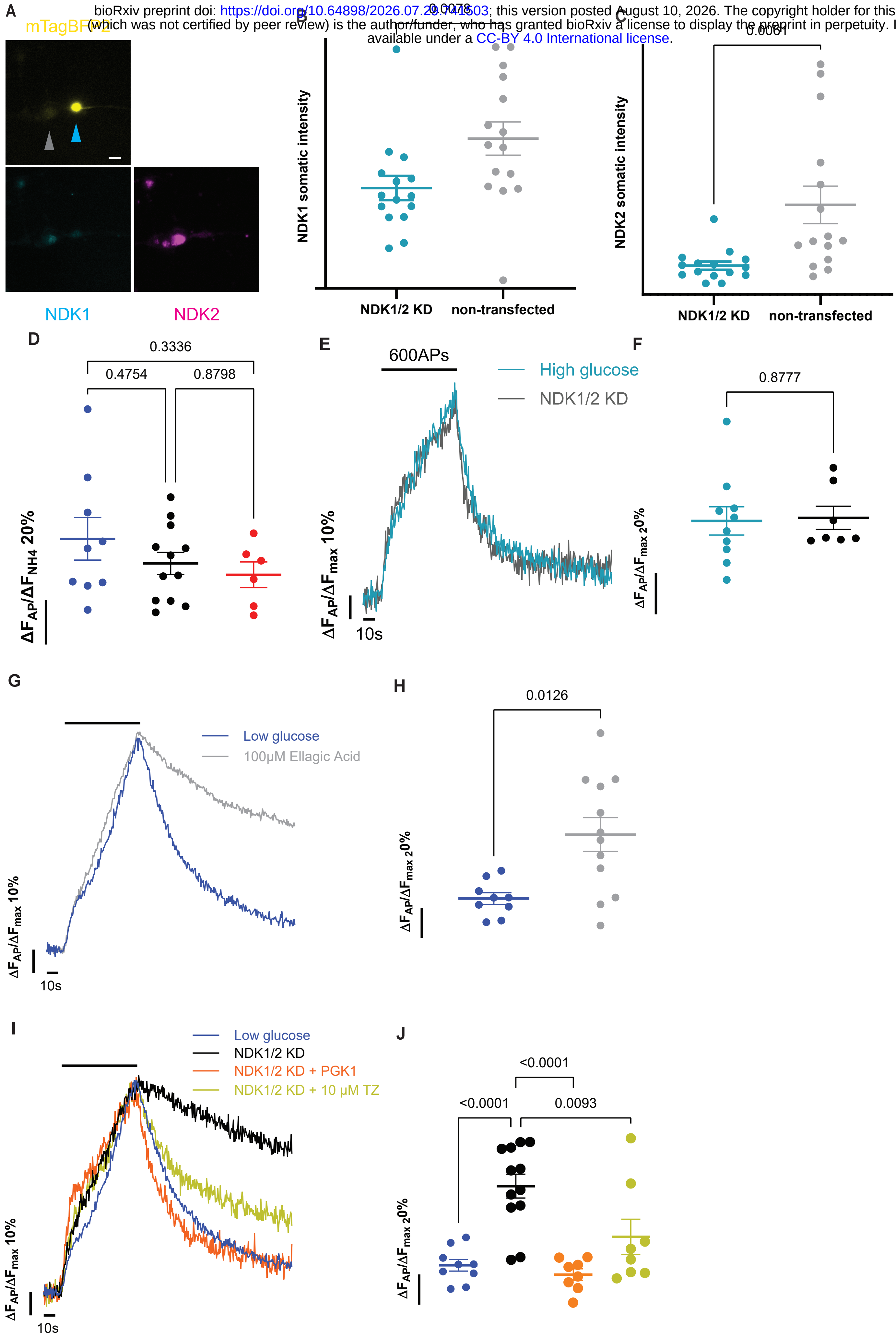

Figure S2

bioRxiv preprint doi: https://doi.org/10.64898/2026.07.29.741503; this version posted August 10, 2026. The copyright holder for this preprint (which was not certified by peer review) is the author/funder, who has granted bioRxiv a license to display the preprint in perpetuity. It is made available under a CC-BY 4.0 International license.

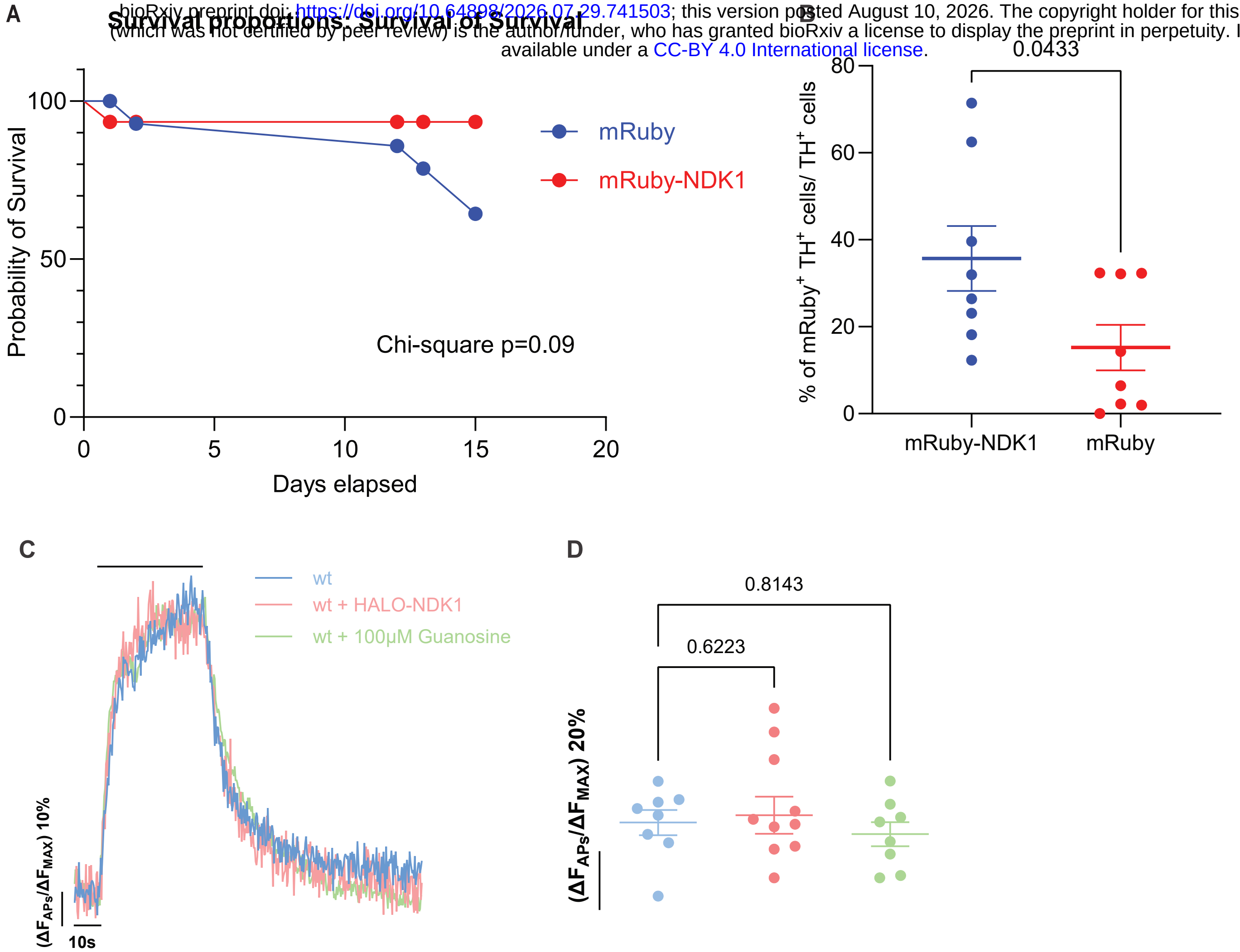

Figure S3

bioRxiv preprint doi: https://doi.org/10.64898/2026.07.29.741503; this version posted August 10, 2026. The copyright holder for this preprint (which was not certified by peer review) is the author/funder, who has granted bioRxiv a license to display the preprint in perpetuity. It is made available under a CC-BY 4.0 International license.

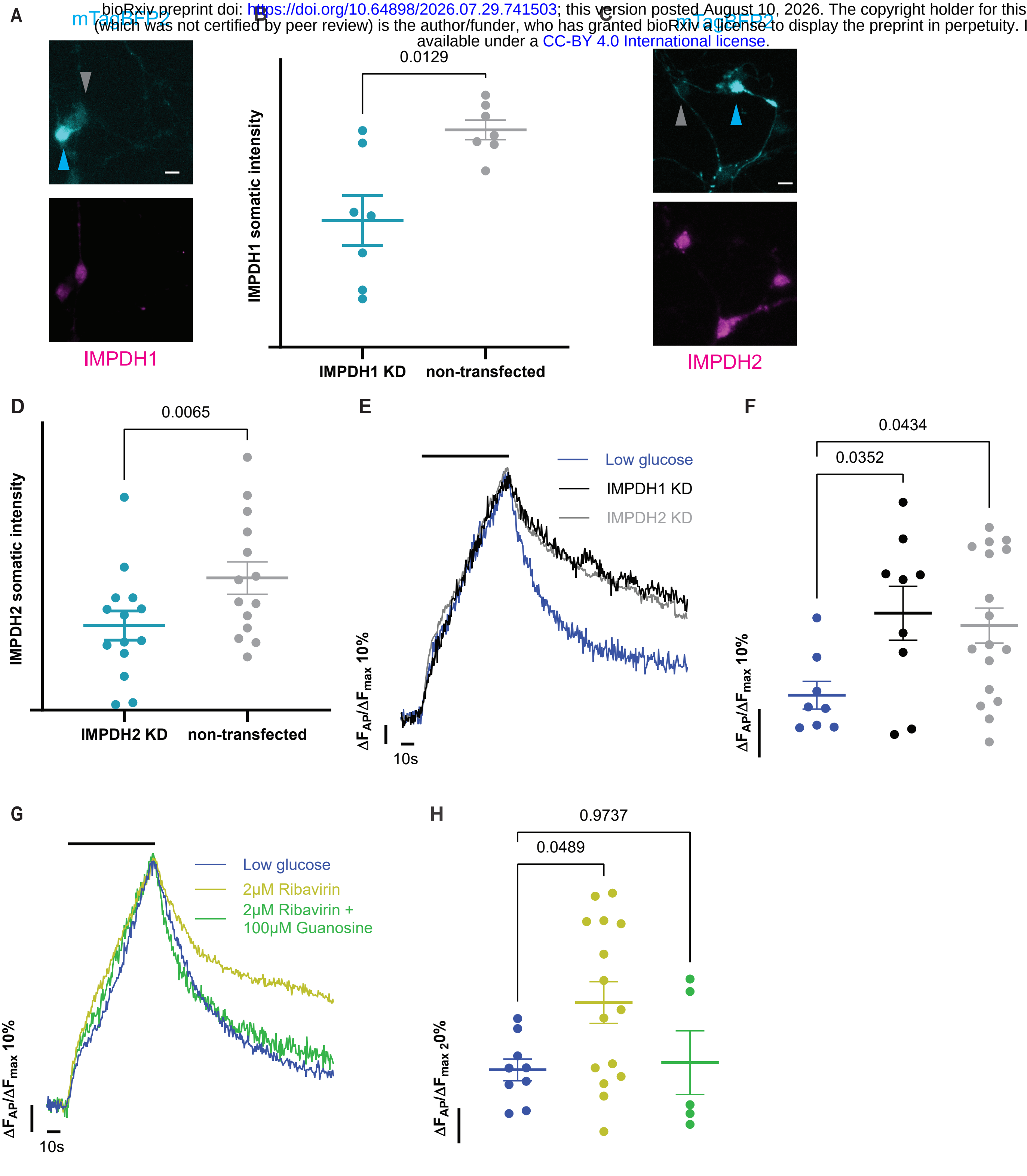

Figure S4

bioRxiv preprint doi: https://doi.org/10.64898/2026.07.29.741503; this version posted August 10, 2026. The copyright holder for this preprint (which was not certified by peer review) is the author/funder, who has granted bioRxiv a license to display the preprint in perpetuity. It is made available under a CC-BY 4.0 International license.

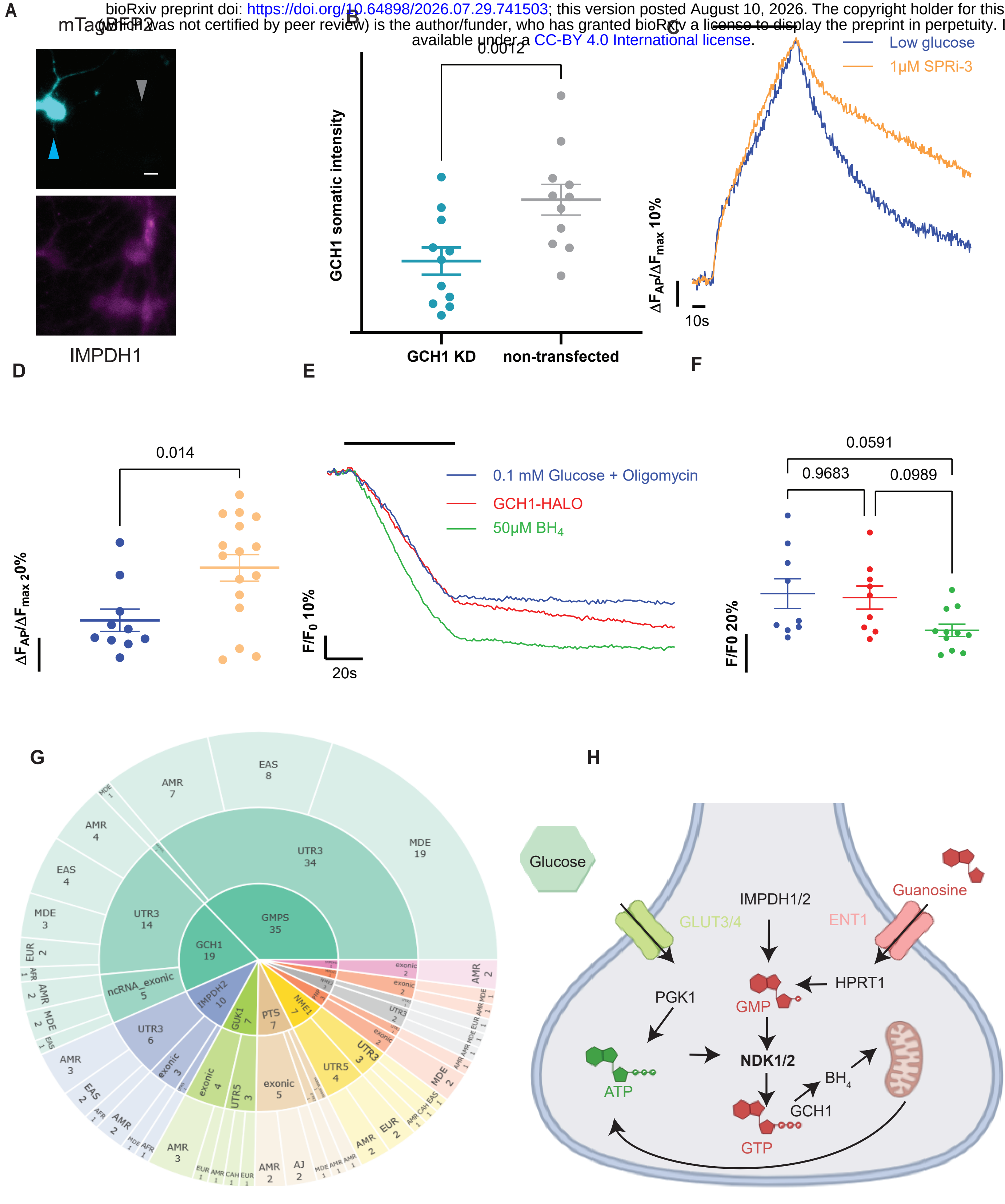

Figure S5
